# Prenatal detection of chromosome 7q deletion with duplication: A case report and literature review

**DOI:** 10.1097/MD.0000000000038461

**Published:** 2024-06-07

**Authors:** Jinping Zhu, Juan Hu

**Affiliations:** a Genetic Medical Center, Women and Children’s Hospital of Linyi City, Liyin, China.

**Keywords:** chromosomal microarray analysis, chromosome 7q deletion/duplication, genome copy number variation, NIPT, prenatal diagnosis

## Abstract

**Rationale:**

With advances in prenatal diagnostic techniques, chromosomal microdeletions and microduplications have become the focus of prenatal diagnosis. 7q partial monosomy or trisomy due to a deletion or duplication of the 7q end is relatively rare and usually originates from parents carrying a balanced translocation.

**Patient concerns:**

Noninvasive prenatal screening (NIPT) showed a fetus with partial deletion and duplication of chromosome 7q. It was not possible to determine whether the fetus was normal.

**Diagnoses:**

Conventional chromosome G-banding and chromosome microarray analysis (CMA) were performed on fetal amniotic fluid samples and parental peripheral blood samples.

**Interventions:**

The pregnant women were given detailed genetic counseling by clinicians.

**Outcomes:**

The fetal karyotype was 46, XY on conventional G-banding analysis. The CMA test results showed a deletion of approximately 7.8 Mb in the 7q36.1q36.3 region and a duplication of 6.6Mb in the 7q35q36.1 region. The parents’ karyotype analysis and CMA results were normal, indicating a new mutation.

**Lessons:**

CMA molecular diagnostic analysis can effectively detect chromosomal microdeletions or microduplications, clarify the relationship between fetal genotype and clinical phenotype, and provide a reference for prenatal diagnosis of chromosomal microdeletion-duplication syndrome.

## 1. Introduction

With advances in prenatal diagnostic techniques, chromosomal microdeletions and microduplications have become the focus of prenatal diagnosis. 7q partial monosomy or trisomy due to a deletion or duplication of the 7q end is relatively rare and usually originates from parents carrying a balanced translocation. The clinical presentation is highly variable due to the different chromosomal regions involved. The main clinical phenotypes of 7q terminal deletion include structural abnormalities of the brain and face, developmental delay, intellectual disability, limb abnormalities, and sacral abnormalities.^[1,2]^ Terminal duplication of 7q is mainly manifested as macrocephaly, prominent forehead, small nose, low-set ears, and developmental delay.^[3,4]^In this study, we combined karyotyping and chromosomal microarray analysis (CMA) to analyze the genetic characteristics of a fetus with partial deletion and duplication of chromosome 7 terminal detected by noninvasive prenatal screening (NIPT), and to provide a reference for clinical genetic counseling. Presently reports as follows:

## 2. Case report

### 2.1. Subjects

Pregnant woman, 37 years old, 3 pregnancies and 1 delivery, G1 delivered a healthy boy at full term, G2 had an abortion at 60 days of gestation due to embryonic failure, and G3 had an abortion during the current pregnancy. Attended our prenatal diagnostic center for NIPT suggesting a 7.8 Mb deletion of chromosome 7 q36.1q36.3 and a duplication of 9.3 Mb in the region of 7q34q36.1. Fetal systemic ultrasound suggests a cystic area in the midline of the fetal brain and a cystic area in the fetal right orbit. Amniocentesis was performed 19^+2^ days. The couple is physically fit and denies consanguineous marriage, has no family history of hereditary disease, no history of infectious disease, no history of exposure to radioactivity, no history of special medications, no history of diabetes or hypertension, and no infections during pregnancy. All examinations were approved by the Ethics Committee of Linyi Maternal and Child Hospital (No. KYL-YXLL-2022017), and the pregnant woman signed the informed consent form.

### 2.2. Cytogenetic analysis

Under ultrasound localization and guidance, amniocentesis was performed through the abdominal wall and 30 mL of amniotic fluid was withdrawn, of which 10 mL was used for CMA. The remaining 20 mL was centrifuged and inoculated into an amniotic fluid medium for 2-lineage culture, and the amniotic fluid cell culture was applied for chromosome preparation by digestion. Parents’ peripheral blood was extracted 2 mL for lymphocyte culture and chromosome preparation, and chromosomes were analyzed at the level of 320 to 400 bands. Karyotyping was performed according to the International System for Human Cytogenetic Nomenclature Guidelines (ISCN2020), with 20 counts per case and 5 mid-division phases analyzed. An additional 10 mL was used for genomic DNA extraction.

### 2.3. CMA testing

Genomic DNA was extracted from 10 mL of fetal amniotic fluid and 2 mL of parental peripheral blood using the Tiangen DNA extraction kit. Genome-wide assays were performed using the CytoScan@750K chip (containing 20,0000 single nucleotide polymorphisms [SNP] probes and 55,0000 copy number variation [CNV] probes) provided by Affymetrix, USA. Experimental methods are in strict accordance with the manufacturer instructions. Several steps including digestion, ligation, PCR, purification, labeling, hybridization, and scanning were applied to the Chromosome Analysis Suite v4.0 software for the analysis of SNP and CNV. The discovered CNVs were searched and interpreted in conjunction with international public databases of genomes and phenotypes, including DECIPHER, OMIM, DGV, UCSC, ClinGen, and PubMed. Pathogenicity analysis and descriptive diagnosis were performed according to the American Society for Medical Genetics and Genomics Sequence Variant Classification Criteria and Guidelines 2019.

**Figure 1.**
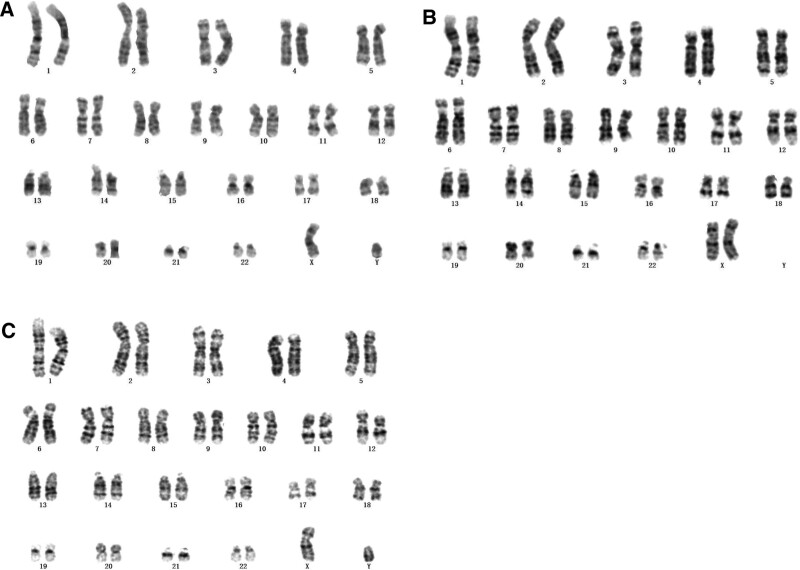
Results of karyotype analysis of the fetus and the parents. (A) Fetal amniotic fluid cell karyotype; (B) the mother peripheral blood chromosome karyotype; (C) Chromosome karyotype of the father peripheral blood.

The Results of routine G-banding chromosome analysis showed that the child had a karyotype of 46, XY, as shown in Figure 1A. Both parents had normal karyotypes (Fig. 1B and C).

**Figure 2.**
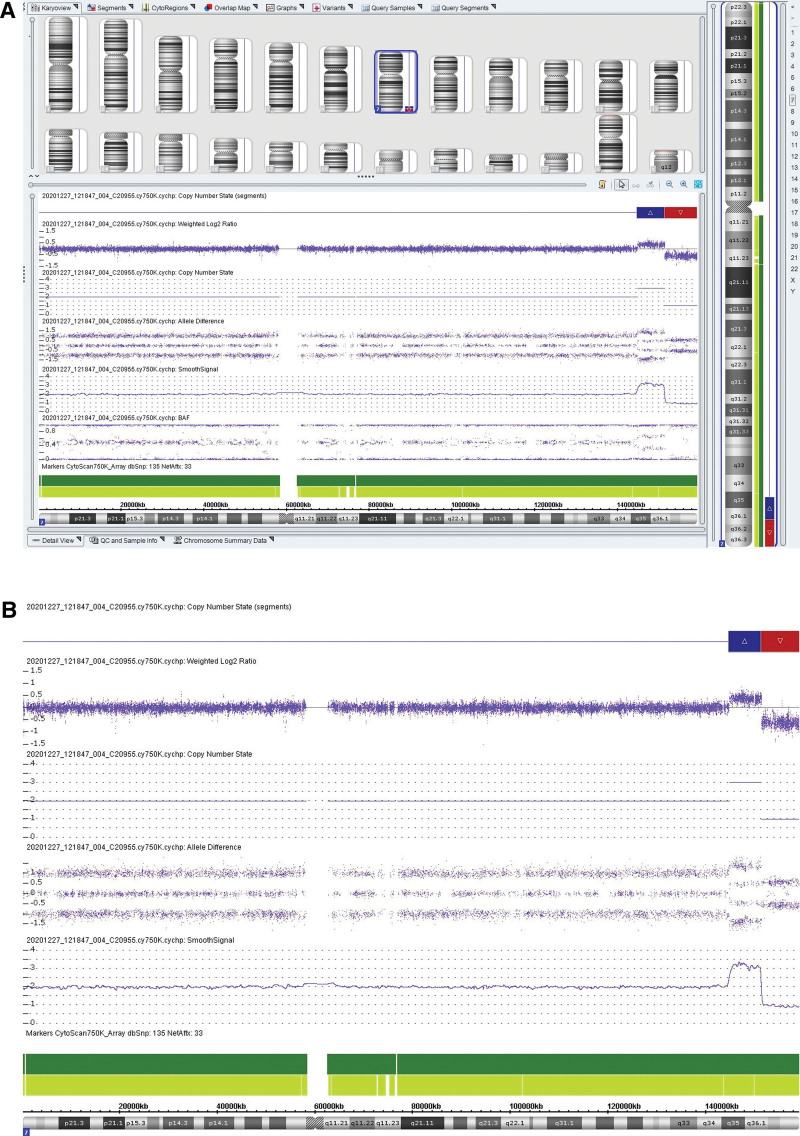
Fetal CMA results. (A) CMA results of fetal chromosome 7; (B) Enlarged view of the long arm of chromosome 7; The red region represents the 7q36.1q36.3 deletion region and the blue region represents the 7q35q36.1 duplication region. CMA = chromosome microarray analysis.

The results of CMA showed that arr[hg19] 7q36.1q36.3(151,262,063-159,119,707) × 1, arr[hg19] 7q35q36.1(144,652,788-151,252,408) × 3, (See Fig. 2A and B). There was a copy number deletion of 7.8Mb in q36.1q36.3 region and a copy number duplication of 6.6Mb in q35q36.1 region in this sample. The q36.1q36.3 region contained 25 OMIM genes, including KMT2C, DPP6, EN2, SHH, MNX1, etc. The q35q36.1 region contained 47 OMIM genes such as CNTNAP2 and KCNH2. No CNV was found in the parents.

Fetal ultrasound showed a 0.9 cm*0.5 cm cystic area posteriorly superior to the third ventricle at the midline of the fetal brain, and a 0.5 cm*0.3 cm cystic area posteriorly to the eyeball in the right orbit. Combined with the results of ultrasound examination, karyotype analysis and CMA results, the pregnant woman and her relatives chose to terminate the pregnancy after genetic counseling. The appearance of the induced fetus was normal, and the pregnant woman refused autopsy.

## 3. Discussion

The clinical phenotypes of 7q36 deletion syndrome are complex and variable. For the 7q36 terminal deletion, submicroscopic abnormalities could hardly be detected without the use of molecular genetic techniques. So far, there are limited clinical cases of 7q36 deletion syndrome. To better characterize the interpretation of the 7q36 microdeletion, we summarized the cases of patients with a pure 7q36 deletion based on the literature review, as shown in Table 1. As can be seen from Table 1, all 7q36 microdeletions reported in the literature vary in size, from 1.2 Mb to 10.4 Mb, patient ages range from fetal to 13 years, and phenotypes range from severe malformations to mild abnormalities, with or without intellectual disability. As can be seen from Table 1, all 7q36 microdeletions reported in the literature vary in size, from 1.2 Mb to 10.4 Mb, patient ages range from fetal to 13 years, and phenotypes range from severe malformations to mild abnormalities, with or without intellectual disability. Except for the unknown genetic mode, the microdeletions of all patients were de novo mutations. The clinical phenotype is complex and diverse, because the size and location of the missing regions are different, and may be related to various functions and sensitive genes. There were 3 haploinsufficiency-sensitive genes in this region, including SHH (#OMIM: 600725), MNX1 (#OMIM:142994), and KMT2C (#OMIM: 606833). The hedgehog protein encoded by the SHH gene plays an important role in the development and regulation of the early formation of the brain, limbs, spinal cord, and teeth.^[21]^ SHH gene mutation or deletion can lead to holoprosencephaly type 3, corpus callosum agenesis with microphthalmia, and maxillary central incisor, which is evaluated as pathogenic. There were 3 cases of holoprosencephaly, 3 cases of corpus callosum agenesis,3 cases of ocular abnormalities, and 8 cases of single maxillary central incisor. In this case, fetal systemic ultrasound showed a cystic area in the midline of the fetal brain and a cystic area in the right orbit, which may be related to the deletion of the SHH gene. Variation or deletion of MNX1 can cause Currarino syndrome (presacral mass, anorectal malformation, and sacral agenesis) and is evaluated as pathogenic. Among them, 8 cases showed sacral agenesis. KMT2C gene mutation or deletion can lead to intellectual disability and seizures, which is evaluated as pathogenic. 4 cases showed definite intellectual disability. Among the 23 cases, 13 cases had microcephaly, which may be related to the deletion of the DPP6 gene.^[22]^ With the exception of cases 15, 16, and 17, all the remaining cases showed different degrees of facial deformity. The fetus in this study had a 7.8Mb deletion at the 7q36.1q36.3 region. According to the previous reports, all the deletions involving 7q36 overlapped or partially overlapped with the cases described in our report. Although the fetal ultrasound, in this case, indicated a cystic area in the midline of the fetal brain and a cystic area in the right orbit of the fetus, the phenotype involved in our case did not appear, which may be related to the smaller gestational age.

**Table 1:** Clinical features of previously published literature and present cases with pure microdeletion at 7q36.

Case	Sex/Age	Cytogenetic location	Size of deletion (Mb)	Inheritance	Karyotype	Chromosome microarray results (hg19)	Clinical manifestation	References
1	F/+3 mo	7q36.1-qter	-	De novo	46,XX,del(7) (q36.1-qter).	-	Microcephaly; facial deformity; partial corpus callosum agenesis; psychomotor retardation	Frints SG et al^[2]^
2	M/11 yr	7q36.1	1.2	De novo	46,XY	chr7:148087489-149297261	Facial deformity; learning disability; intellectual disability; developmental delay;	Suri T et al^[5]^
3	M/13 yr	7q36.1-q36.3	6.89	De novo	46,XY,del(7)(q36)	chr7:149120973-155944978	Microcephaly; intellectual disability; seizures; facial deformity; developmental delay; short stature; strephenopodia; penoscrotal transposition; scoliosis	Hyohyeon C et al^[6]^
4	M/-	7q36.3	2.7	De novo	46,XY	chr7:156658622-156816815	Microcephaly; facial deformity; single maxillary central incisor; ear anomalies; ocular anomalies	Coutton C et al^[7]^
5	F/9 yr	7q36.1-q36.2	5.27	De novo	46,XX	chr7:148262352-153562352	Intellectual disability; feeding problems; renal dysgenesis; long QT syndrome; facial deformity	Caselli R et al^[8]^
6	F/10 yr	7q36-qter	-	De novo	46, XX, del(7)(q36 − qter)	-	Facial deformity; hypotonia; feeding problems; developmental delay; sacral agenesis; arcuate uterus	Su PH et al^[9]^
7	M/13 yr	7q36.2q36.3	2.9	De novo	46,XY,del(7) (q36.1-q36.3)	Chr7:153,206,357–156,133,135	Developmental delay; facial deformity; short stature; microcephaly; maxillary central incisor; finger deformity; ocular anomalies	Beleza-Meireles et al^[10]^
8	M/37 mo	7q36.3	-	De novo	46,XY,del(7)(q36.1)	-	Developmental delay; facial deformity; partial sacral agenesis; microcephaly;tethered cord	Horn D et al^[11]^(case 1)
9	M/3 yr, 6 mo	7q36.3	-	De novo	46,XY, del(7)(q36.3)	-	Developmental delay; facial deformity;single maxillary central incisor;corpus callosum agenesis； pituitary gland hypoplasia microcephaly	Horn D et al^[11]^(case 2)
10	F/13 yr	7q36.2q36.3	6.6	De novo	46,XX,del(7)(q36)	Chr7:152582067-159125239	Microcephaly; facial deformity; moderate hearing problems; hypotonia; psychomotor retardation; partial sacral agenesis	Ayub S et al^[12]^
11	M/18 mo	7q36-qter	-	De novo	46,XY del (7)(q36–qter)	-	Developmental delay; intellectual disability; sacral agenesis; microcephaly; hydrocephaly; hypospadias; anterior myelomeningocele; facial deformity; ocular anomalies	Rodríguez L et al^[13]^
12	F/12 yr	7q36.1-qter	-	De novo	46,XX,del(7)(pter-q36.1:)	-	Developmental delay; intellectual disability; microcephaly; sacral agenesis; single maxillary central incisor; facial deformity; ectopic kidneys; double renal pelvis	Masuno M et al^[14]^(case 1)
13	M/12 yr	7q36.1-qter		-	46,XX,del(7)(pter-q36.1:)		Developmental delay; mental retardation; microcephaly; facial deformity; single maxillary central incisor, scoliosis; hearing loss; bilateral hydroureteronephrosis; sacral occulta spina bifida	Masuno M et al^[14]^(case 2)
14	Fetal	7q36-qter	-	-	46,XX, del(7) (q36)		Developmental delay; facial deformity; polyhydramnios; semilobar holoprosencephaly; bilateral hydronephrosis;	Benzacken B et al^[15]^(case 1)
15	F/-	7q36-qter	-	-	46,XY,del(7)(q36-qter)	-	Holoprosencephaly	Roessler E et al^[16]^(case 1)
16	M/-	7q36-qter			46,XX,del(7)(q36-qter)		Developmental delay; microcephaly	Roessler E et al^[16]^ (case 2)
17	M/-	7q36-qter			46,XY,del(7)(q36-qter)		Short stature	Roessler E et al^[16]^ (case 3)
18	F/3 yr	7q36.2q36.3	4.15	-	-	chr7:153045998-157128785	Currarino syndrome (presacral mass, anorectal malformation and sacral dysgenesis); microcephaly	Cococcioni L et al^[17]^
19	M/2 yr	7q36-7qter	-	-	46,XY,del(7)(q36-qter)	-	Microcephaly; syringomyelia (C4 and D4); craniovertebral hinge abnormalities; butterfly vertebral (L5); hirschsprung; single maxillary central incisor; talipes	Lami F et al^[18]^(case 2)
20	F/7 yr	7q36.2- 7q36.3	5.6	-	46,XX,del(7)(q36-qter)	chr7:153669067-159107239	Facial deformity; solitary maxillary central incisor; cerebellar vermis hypoplasia; mild ventricular dilatation; anterior sacral meningocele; partial sacral agenesis; sacral vertebra cleft	Lami F et al^[18]^(case 3)
21	Fetal	7q36.2- 7q36.3	-	-	46,XN,del(7)(q36-qter)	-	Intrauterine growth retardation; corpus callosum agenesis; ventricular septal defect; biventricular aorta; single umbilical artery; omphalocele	Lami F et al^[18]^(case 4)
22	M/12 yr	7q36.1-qter	10.02	De novo	46,XY,del(7)(q36.1)	chr7:149165115-159128555	Developmental delay; language retardation; partial sacral agenesis; intellectual disability; dental developmental abnormalities; hypospadias	Linhares ND et al^[19]^
23	M/18 mo	7q36.1- 7q36.3	10.4	-	-	chr7:148719655-159119707	Facial deformity; psychomotor retardation;	Xi Hongmin et al^[20]^

- = Unknown, F = female, M = male.

7q distal duplication is rare, and the phenotype varies depending on the duplication region. Kerri Bosfield et al summarized the clinical phenotype of 15 patients with distal duplication of 7q and found that their common functional abnormalities included global developmental delay, frontal bulge, macrocephaly, seizures, kyphosis/skeletal abnormalities, mandibular retrusion/palatal abnormalities and other symptoms.^[23]^In this study, the fetus had about 6.6 Mb duplication in the 7q35q36.1 region, and no identical cases were found at present. No microduplication syndrome in this region was found in the OMIM database. Only Piero Pavone et al reported a case of a 7q36 deletion with a 7q34q35 duplication involving SHH, MNX1, and EN2 genes. This case presented with Currarino syndrome (anorectal malformation, presacral tumor, and sacral agenesis), developmental delay, microcephaly, neurological deafness, intellectual disability, cerebellar vermis hypoplasia, and growth hormone deficiency.^[24]^ Therefore, the results of this study enrich the genotype information of 7q distal duplication and provide a basis for clinical diagnosis of 7q duplication and deletion.

Conventional karyotyping cannot detect a difference of <10 Mb. In this study, conventional karyotyping did not find obvious abnormalities in the karyotype of the fetus, especially the microduplication and microdeletion. The location and length of 7q deletion or duplication are not fixed, so the clinical phenotypes and disease conditions of patients with this variation are quite different. However, with the application of gene chip and next generation sequencing, there is a trend to refine chromosomal breakpoint analysis, evaluate key regions related to genetic diseases, and reveal small rearrangements that cannot be obtained by traditional methods. However, there is no effective treatment for chromosome microdeletion/microduplication syndrome. Therefore, genetic counseling and prenatal diagnosis can effectively reduce the birth of children with chromosomal microdeletion/microduplication syndrome, and reduce the burden on families and society.

## Author contributions

**Conceptualization:** Jinping Zhu.

**Data curation:** Jinping Zhu, Juan Hu.

**Formal analysis:** Jinping Zhu.

**Funding acquisition:** Jinping Zhu.

**Investigation:** Jinping Zhu.

**Methodology:** Jinping Zhu.

**Project administration:** Jinping Zhu.

**Resources:** Jinping Zhu.

**Writing – original draft:** Jinping Zhu.

**Writing – review & editing:** Jinping Zhu, Juan Hu.
